# Interventions Change Soil Functions and the Mechanisms Controlling the Structure of Soil Microbial Communities

**DOI:** 10.3390/microorganisms11061502

**Published:** 2023-06-05

**Authors:** Effimia M. Papatheodorou

**Affiliations:** Department of Ecology, School of Biology, AUTH, 54124 Thessaloniki, Greece; papatheo@bio.auth.gr

Soil microbial communities play essential roles in maintaining ecosystem functions, such as litter decomposition, mineralization, nitrification, and denitrification, thus exerting control on primary production, soil fertility, and gas emissions. Disturbances induced by changes in abiotic and/or abiotic elements of soil habitats due to modifications in land use and climate affect the composition and structure of soil microbial communities, with subsequent changes in ecosystem functions.

However, the relation between microbial community assemblage and a specific function or a weighted index of microbial-mediated functions (multifunctionality index [1]) could be affected by various factors. These could be variables used to describe community organization (functional diversity, species richness, composition, and co-occurrence patterns [2]) and variables that are related to the taxonomic level and spatial scale of the community under consideration [3]. Most studies have explored the relation between taxa richness and functions, while information concerning the relationship between the co-occurrence patterns of microbes reflecting a community structure and the functionality of an ecosystem is rather limited [4]. Additionally, current discussion on microbial communities’ assemblage has moved one step forward and incorporates the idea of stochasticity and determinism. Determinism is related to the niches’ traits of taxa, while stochasticity refers to species’ unlimited dispersal and ecological drift [5,6]. Knowledge of the mechanisms that control microbial communities’ assemblages and their relation to soil functionality could be extremely useful for conservation, restoration, and management efforts.

This Special Issue, entitled “Soil microbial communities and ecosystem functions”, contains four original contributions focusing on the changes in microbial communities’ composition and diversity in relation to soil chemical variables and/or enzyme activities, which are used as proxies of soil functionality, and a review article discussing the pitfalls of soil metagenomics. In the included research articles, microbial communities are considered under conditions of tillage/no tillage [7], organic fertilizers’ legacy [8], continuous cultivation [9], and fire [10]. Importantly, the authors analyzed not only the quantitative aspects of the community under consideration, such as biomass, abundance, and diversity, but they also discussed the qualitative aspects resulting from the network of correlations among the members of the community, such as network connectance, centrality, betweenness, and keystone taxa.

Microbial communities have been extensively studied during the last decade. A quick search in Scopus in April 2023 using the terms “soil” and “microb*” as keywords and limited to the last 10 years yielded a total of 72,138 research articles. The development of next-generation sequencing (NGS) technique jointly with the lowering of sequencing cost has made this technique applicable almost everywhere. However, metagenomics analysis has some drawbacks that are discussed by Leite et al. [11]. The lack of information regarding physicochemical soil environments is reported as one of the major problems. Soil physicochemical properties play a crucial role in determining soil microbiome composition and function [12]. However, most soil microbiome studies either do not include soil physicochemical properties or limit measurements to pH, soil organic carbon or total carbon, and total nitrogen. However, these variables describe the storage of carbon and nitrogen in soil and not their availability. Moreover, the complex soil chemical status is also described by other nutrients, such as P, K, Ca, Mg, Mn, and Fe, which are affected by climate, parent material, land use, and aboveground composition. Since microbial communities depend on their physicochemical environment, the lack of such data means that a source of explanation is missing. Accounting for soil properties can provide a better understanding of which microbes and why these specific microbes are especially sensitive to certain abiotic changes and the potential impact of this sensitivity on their ecosystem function [11]. The second problem mentioned by Leite et al. [11] is the lack of untreated controls. For instance, researchers could examine the effect of different types of fertilizers on microbial community composition, and although researchers have recorded different communities between different fertilizers, they could not identify whether these communities are significantly different from the community in the untreated samples or whether the microbial diversity in the treated plots is lower/higher compared to the untreated ones. The incorporation of a control would create baseline data where specific communities could be identified in specific ecosystems. Without an untreated control, it is very difficult to determine the causes of changes in soil microbial communities. Finally, the third drawback in metagenomics analysis is related to the way of sharing metagenomics data. Although data sharing and data publicity are obligatory for publication in most cases, frequently only a part of data becomes available (a fact that has to do with journals’ policies), or the metadata that characterize each group of data and contain information about the site, soil depth, or other decisive abiotic variables are not provided. Leite et al. [11] declared that only if researchers follow the FAIR (findable, accessible, interoperable, reusable) guidelines for scientific data, the reliability in the meta-analysis of data and the reproducibility of scientific research will be ensured.

## Interventions, Soil Functions, and Microbial Community Assemblage

The use of tillage in croplands, compared to grasslands where no tillage is applied, induces changes in the composition but not the diversity of soil bacterial communities [7]. In grasslands, there is much more dependence of microbial community composition on soil nutrients, demonstrating that the composition of bacterial communities in these soils is more susceptible to environmental variables than in cropland soils. The latter fits well with the findings that microbial community assemblage in grasslands is mostly under deterministic regulation, while in croplands, the regulation is much more stochastic. One of the mechanisms of stochastic regulation is unlimited dispersal [5]. It was found that proper tillage applied to croplands resulted in the homogeneity of soil environment, which permitted bacteria to exhibit a high migration rate. Furthermore, tillage increased the network connectivity and complexity of cropland bacterial community but decreased its compactness.

Biochar and compost are beneficial organic amendments used in sustainable agriculture; they are both produced by the treatment of agricultural wastes following different procedures. However, according to Xin et al. [8], only biochar exhibited a legacy effect on the composition and diversity of the community of arbuscular mycorrhizal fungi (AMF). Soil available N, P, and total P were the most important predictors for AM fungal Shannon diversity, which was enhanced by biochar application. Additionally, the incorporation of biochar simplified the AM fungal co-occurrence network and decreased the network size, while most interactions between arbuscular mycorrhizal fungi were positive, thus revealing cooperation as the main interaction type [8].

The response of soil bacterial and fungal communities and of soil biochemical variables to long-term continuous monocropping (from 5 to 20 yrs) of water oats was examined by Wu et al. [9]. The soil biochemical properties and the activity of soil enzymes were higher at 5 to 10 yrs of cultivation and then declined. The long-term continuous planting of water oats significantly reduced the richness of the operational taxonomic units and the diversity indices of rhizosphere bacteria and fungi. Although the abundance of bacteria appeared independent on the duration of cultivation, significant changes in community composition were recorded. For fungi, continuous monocropping induced changes in community composition and abundance. For both microbial groups, the changes in their communities were related to soil fertility (available P, K, N, and soil organic carbon).

Among human interventions, fire is one of the most common in Mediterranean areas; its effects on the assembly rules of soil prokaryotes in a small-scale (rhizosphere, bulk soil) environment have been studied [10]. The values of the networks’ parameters produced by the co-occurrence patterns of soil bacteria were used to describe the assembly rules and the communities’ robustness against disturbances. Although the networks in the burnt areas contained more co-occurring taxa, were less fragmented, had lower null ties, exhibited stronger local organization, and had larger neighborhoods compared to the networks in unburnt soils, the most diversified were the unburnt rhizosphere and the burned bulk soil network. The mechanisms mainly responsible for bacterial community structure were stochastic in all soils, either burnt or unburnt, and the contribution of stochasticity differed between rhizosphere soils; the communities in burnt rhizospheres were much more stochastic than in unburnt rhizospheres.

**Figure 1:**
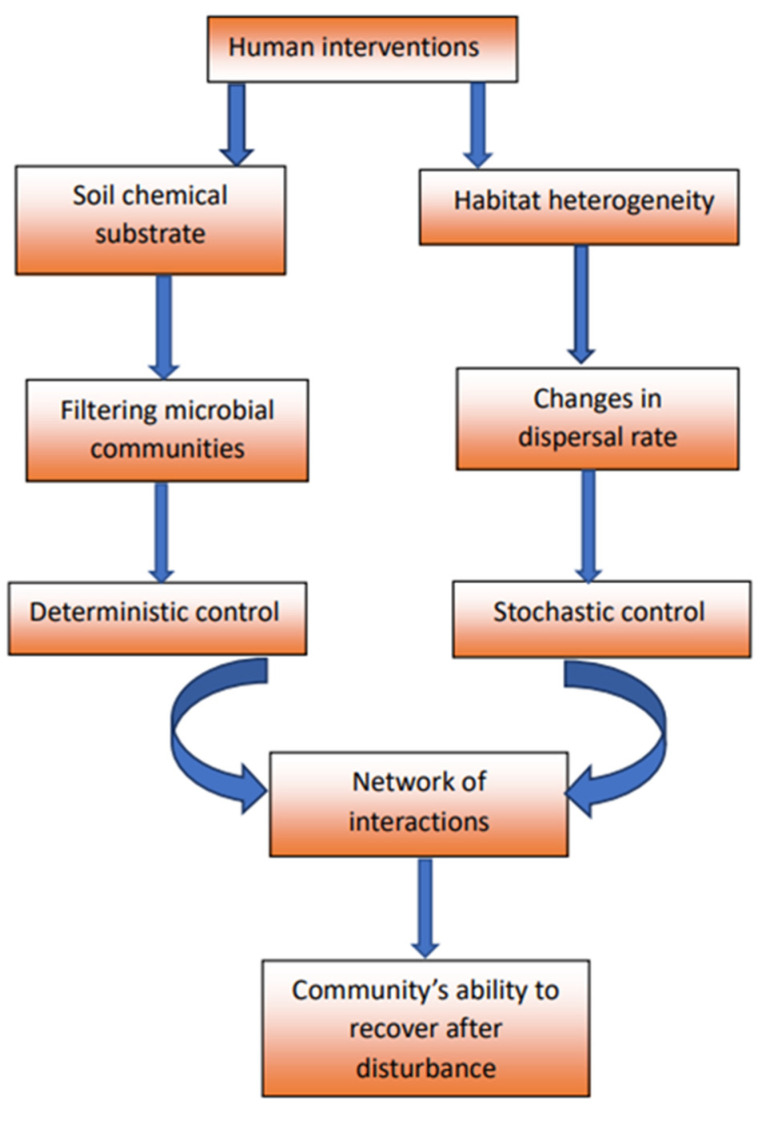
Human interventions induce changes in soil environment, thereby altering the mechanisms that control soil microbial community assemblage. This is imprinted on the network of interactions among microbial members and determines a community’s ability to recover after disturbances.

Different human interventions (tillage, continuous monocropping, organic amendments’ application, and fire) induce changes in soil chemical background that filter the composition of microbial communities. Such interventions also affect the heterogeneity of microbial habitats and, consequently, the dispersal rate of microbes; the latter has an impact on the mechanisms that control community assemblages (stochasticity and determinism). This is further imprinted on the network of interactions among community members, which provide valuable insights into these communities’ ability to recover after disturbances ([13,14,15]; the above are summarized in Figure 1).
